# Magnetic Resonance Image in Monitor and Diagnosis of Patients with Neuromyelitis Optica

**DOI:** 10.1155/2022/1430380

**Published:** 2022-03-17

**Authors:** Shujuan Lu, Dongming Wang, Fengxian Zhang, Meilan Liu

**Affiliations:** ^1^Department of Imaging, Shanting District People's Hospital, 528 Xicheng Beijing Road, Shanting District, Zaozhuang 277200, Shandong, China; ^2^Department of CT/MRI, Tengzhou Traditional Chinese Medicine Hospital, No. 52 Shangguozhong Road, Tengzhou, Zaozhuang 277500, Shandong, China; ^3^Department of Imaging, New Hospital District of People's Hospital of Sishui County, The Intersection of Shenghua Road and Quan Road, Sishui County, Jining 273200, Shandong, China; ^4^Department of Imaging, New Hospital District of Zaozhuang Traditional Chinese Medicine Hospital, 2666 Taihang Mountain Road, Xuecheng District, Zaozhuang 277100, Shandong, China

## Abstract

This study was aimed to investigate the craniocerebral magnetic resonance imaging (MRI) measurement and clinical characteristics of patients with neuromyelitis optica (NMO) and multiple sclerosis (MS). 50 patients with NMO (NMO group) and 50 patients with MS (MS group) were studied. The clinical manifestations, brain injury morphology, MRI signal characteristics, brain distribution characteristics, and related laboratory tests (serum aquaporin 4 [AQP4] antibody) were statistically analyzed. The results showed that the analysis of clinical manifestations of patients revealed that optic neuritis (37 cases) was the most common disease in NMO patients, and myelitis (16 cases) was more common in MS patients than NMO patients. There were significant differences in gender ratio, abnormal expression of brain MR1, positive serum AQP4-IgG, and other immune diseases and symptoms between the two groups (*P* < 0.05). When the lesions measured by MRI were located in the white matter area of the cerebral hemisphere, the image showed blurred edges and a relatively symmetrical distribution. When the lesions measured by MRI were located around the medulla oblongata, the lesions mainly involved the central gray matter and white matter of the spinal cord, with patchy and line-like long T1 and long T2 signals. Moreover, in the late stage of recurrence of spinal cord disease, severe spinal cord atrophy may occur. In conclusion, craniocerebral MRI measurement in NMO patients can provide more basis for the diagnosis and differential diagnosis of the disease, so as to improve the diagnostic level of NMO.

## 1. Introduction

Neuromyelitis optica (NMO), also known as Devic's disease, is an immune-mediated, demyelinating disease in which acute or subacute optic nerve or spinal cord is affected simultaneously or sequentially [1]. Given the high incidence in Asian and African populations, there have been divergent views on whether NMO is a subtype of MS or an independent disease. In recent years, NMO, which is a more specific aquaporin 4 (AQP_4_) antibody, separates NMO from multiple sclerosis (MS) [2, 3]. NMO differs from MS in the distribution, immunity, pathological changes, clinical and imaging changes, and treatment of east and west races, so it is of great significance to identify NMO and MS in the early stages of the disease. In addition, MRI has been used for clinical diagnosis since its introduction in the 1980s.

In recent years, the widespread application of MRI measurements is of great significance for the early diagnosis and early treatment of NMO patients. MRI is more sensitive to the abnormal signal display of the brain and spinal cord, especially the T2-weighted sequence can clearly show its signal changes. At the same time, MRI's multiplanar and multiparameter imaging technology can clearly show the location, size, and relationship with surrounding tissues of NMO brain and spinal lesions [4, 5]. With the continuous development of new magnetic resonance (MR) technology, many scholars have applied sensors to magnetic resonance imaging (MRI). MRI will be further improved. Ziem et al. (2019) used the new magnetic induction mode of quantum sensor or nanoprobe to greatly improve the sensitivity of MRI, thus improving the nanospatial resolution. By optimizing the control spin operation, in the nonuniform control field, the thin-layer ensemble spin sensor was used to enhance the multichannel MRI to improve the ensemble sensitivity and field of view (FOV) [6]. Miller et al. (2021) used innovative physicochemical mechanisms to improve sensors and make MRI acquisition more sensitive [7]. Therefore, MRI combined with sensors is applied to NMO diseases. It can diagnose central demyelinating diseases not only from the perspective of anatomical structure but also from the perspective of pathophysiology, biochemical changes, and histology. Therefore, it plays an increasingly important role in clinical work.

The purpose of this article is to retrospectively analyze various manifestations of the craniocerebral of NMO, to closely combine clinical manifestations and related laboratory examinations, to summarize the more specific signs of NMO patients, and to deepen the clinical, imaging, and related laboratory indicators of NMO. The knowledge helps to diagnose the disease earlier and more accurately. Meanwhile, the imaging measurement results of 50 patients with MS and 50 patients with NMO were summarized and analyzed to study the MRI findings and the relationship between them, and summarize the intracranial MRI findings of patients with NMO. The purpose is to provide more basis for the clinical diagnosis, prognosis, and treatment of patients with NMO.

## 2. Materials and Methods

### 2.1. Research Objects

The paper retrospectively analyzed 50 cases of NMO patients (NMO group) and 50 cases of multiple sclerosis patients (MS group) treated in hospital from May 2017 to December 2019. Patients with MS who met the 2010 McDonald diagnostic criteria were included in the control group. NMO patients are 11 to 65 years old, and MS patients are 11 to 58 years old. Brain and spinal cord scans were performed in 50 NMO patients, and enhanced scans were performed in 8 patients. All 50 MS patients underwent craniocerebral scans, and 16 patients underwent spinal MRI. The study had been approved by the ethics committee of the hospital, and all the research objects included in this study signed the informed consent forms.

Inclusion criteria: all patients met the 2006 Wingerchuk diagnostic criteria [8], and the prerequisites are optic neuritis and transverse myelitis, and at least two of the three support criteria are met at the same time: (1) spinal MRI lesions are longer than 3 spinal segments; (2) MRI measurement of the brain showed normal or did not meet the diagnostic criteria of MS; and (3) AQP_4_ antibody is positive.

Exclusion criteria: (1) the diagnostic results of patients do not match the NMO patient group; (2) the patient is complicated with other diseases of the central nervous system, such as cerebrovascular disease, intracranial inflammatory diseases, brain trauma, and other diseases affecting the structure of the brain and spinal cord; (3) the patient cannot adapt to the scanning environment and noise, there are uncorrectable head movements during the scanning process, and any of the patient's clinical, imaging, and serological data are incomplete.

### 2.2. Analysis of Brain MRI Image Measurement

The clinical data, MRI image measurement, and related laboratory examination contents of each NMO and MS patients included in the study were retrospectively analyzed. The brain MRI signs were analyzed by blind method. The images were read by two senior imaging doctors. The results are the same. When there are different results, the two doctors will analyze and discuss them together and reach a consensus. The clinical characteristics of patients (onset, first clinical manifestation, visual field disturbance, craniocerebral involvement (hiccups, nausea and vomiting, epilepsy, and disturbance of consciousness), and the characteristics of MRI images (number of lesions, signal characteristics, and enhancement characteristics)) are observed.

### 2.3. Basic Principles of Magnetic Resonance

Nuclear magnetic resonance is a physical process in which atomic nuclei with nonzero magnetic moments undergo Zeeman splitting of spin levels under the action of an external magnetic field, which absorbs radiofrequency radiation at a certain frequency. Nuclear magnetic resonance spectroscopy is a branch of spectroscopy, whose resonance frequency is in the radiofrequency band, and the corresponding transition is the transition of nuclear spins at the nuclear *Caiman* level [9, 10].

Nuclei with nonzero spin quantum numbers interact with the external magnetic field B0, causing the nuclear energy level to undergo a 2I + 1 split, which is called a *Caiman* split.

The difference in energy between two levels at which nuclear spin transitions can occur

(1) $$\begin{array}{c} E=-\mu_{z}B_{0}=-\gamma \cdot m\cdot \hbar \cdot B_{0},\\ \Delta E=\gamma \cdot \Delta m\cdot \hbar \cdot B_{0}. \end{array}$$

If the above static magnetic field B0 is present and a direction perpendicular to it is added, the intensity of the radiofrequency alternating magnetic field B1 is much lower than B0, and its frequency satisfies the following conditions:

(2) $$\begin{array}{c} h\upsilon =\Delta E=\gamma \cdot \hbar \cdot H_{0}. \end{array}$$

The atomic nucleus absorbs the energy of the radiofrequency field and a transition occurs between the two *Caiman* levels. This phenomenon is a nuclear magnetic resonance phenomenon. The resonance frequency is calculated by the following formula:

(3) $$\begin{array}{c} \upsilon =\frac{\gamma \cdot B_{0}}{2\pi }. \end{array}$$

The NMR frequency varies from nucleus to nucleus. For the same nucleus, the resonance frequency is proportional to the static magnetic field B0.

Due to the different chemical environments of the same nucleus in the molecule, the phenomenon of shifting the resonance frequency is called chemical shift. The reason for the chemical shift is that the electrons moving in the molecule magnetically shield the nucleus from the external magnetic field. The shielding effect can be expressed by the shielding factor *σ*, which is always much smaller than 1. Generally, the shielding factor *s* is a second-order tensor. Only in the liquid due to the rapid rolling of the molecules, the anisotropy of the chemical shift is averaged, and the shielding factor appears as a constant. The magnetic field strength felt by the nucleus is as follows:

(4) $$\begin{array}{c} B_{N}=B_{0}\left(1-\sigma \right). \end{array}$$

NMR resonance frequency is calculated by the following formula:

(5) $$\begin{array}{c} \upsilon =\frac{\gamma \cdot B_{0}\left(1-\sigma \right)}{2\pi }. \end{array}$$

In the actual measurement, the chemical shift is based on the spectral line of a certain reference material, and the other spectral lines are compared with it; that is, the magnitude of the chemical shift is represented by a dimensionless quantity *d*. In the applied magnetic field *H*_0_, the spin nucleus rotates around the spin axis, and the spin axis and the magnetic field *H*_0_ rotate around *H*_0_ at a specific angle, similar to a gyro moving in a gravitational field. Precession frequency (also known as Larmor frequency) is calculated by the following formula:

(6) $$\begin{array}{c} W_{0}=2\pi v_{0}=\gamma H_{0}. \end{array}$$

The spin angular momentum is quantized, and the relationship between the component *P*_*z*_ in the magnetic field direction and the magnetic quantum number (*m*) is *P*_*z*_=*mh*/2*π*, because *m* has 2*l*+1 values, and accordingly, *P*_*z*_ also has 2*l*+1 values, corresponding to this. Magnetic moment of the spin core on the *z* axis is as follows:

(7) $$\begin{array}{c} \mu_{z}=\gamma P_{z}=\gamma mh/2\pi , \end{array}$$

*μ* and *H*_0_ interaction energies are calculated by the following formula:

(8) $$\begin{array}{c} E=-\mu_{z}H_{0}. \end{array}$$

Inserting the format into it yields

(9) $$\begin{array}{c} E=\gamma mh/2\pi u. \end{array}$$

Since *m* is quantized, the *E* value is also quantized. This shows that the spin-like energy in the magnetic field is also quantized.

According to the above formula, in the external magnetic field, the spin nucleus has different energy levels. For example, when an electromagnetic wave with a certain frequency *n* is irradiated to the sample, and *v*=*v*_0_, that is, *hv*=Δ*E*=*γh*/2*πH*_0_, the nucleus can make the transition between the energy levels and produce nuclear magnetic resonance absorption to obtain nuclear magnetic resonance spectrum

(10) $$\begin{array}{c} v=\Delta E/h=\gamma H_{0}/2\pi . \end{array}$$

So, the above formula produces the conditions for NMR.

### 2.4. Serum AQP4 Test

All patients collected blood before using hormones, and the blood coagulated naturally at room temperature for 10–20 minutes, centrifuged for about 20 minutes (2000–3000 rpm), and the supernatant was collected. Detection was performed by enzyme-linked immunosorbent assay (ELISA). The human aquaporin 4 antibody (AQP4 Ab) enzyme-linked immunoassay kit was used.

The specific detection steps are as follows:

1. Dilution and loading of standards: 10 wells of standards are set on the enzyme-coated plate, 100 *μ*l of standards is added to the first and second wells, and standards are added to the first and second wells. Dilute 50 *μ*l of the diluent and mix well; then take 100 *μ*l from the first and second wells and add to the third and fourth wells, and add 50 *μ*l of the standard diluent to the third and fourth wells and mix; in the third and fourth wells, first take 50 *μ*l of each discarded, add 50 *μ*l each to the fifth and sixth wells, and then add 50ul of the standard dilution solution to the fifth and sixth wells and mix well; after homogenization, take 50 *μ*l from each of the fifth and sixth wells and add it to the seventh and eighth wells, and then add 50 *μ*l of the standard dilution solution to the seventh and eighth wells. Add 50 *μ*l to the ninth and tenth wells, and add 50 *μ*l of the standard dilution solution to the ninth and tenth wells. After mixing, take 50 *μ*l each from the ninth and tenth wells and discard. (The sample volume of each well after dilution was 50 *μ*l, and the concentrations were 1200 ng/*L*, 800 ng/*L*, 400 ng/*L*, 200 ng/*L*, and 100 ng/*L)*.
2. Adding samples: Set blank wells (the blank control wells do not add samples and enzyme-labeled reagents, and the rest of the steps are the same), the sample wells to be tested. Add 40 *μ*l of the sample dilution to the wells of the sample to be tested on the enzyme-coated plate and then add 10 *μ*l of the sample to be tested (the final dilution of the sample is 5 times). Add the sample to the bottom of the well of the microtiter plate, try not to touch the wall of the well, and shake gently to mix.
3. Incubation: Seal the plate with a sealing plate and incubate at 37°C for 30 minutes.
4. Dosing: Dilute 30 times concentrated washing solution with distilled water 30 times and set aside.
5. Washing: Carefully peel off the sealing plate film, discard the liquid, and dry it. Fill each well with washing liquid, leave it for 30 seconds, and discard it. Repeat this 5 times and pat dry.
6. Add enzyme: Add 50 *μ*l of enzyme-labeled reagent to each well, except for blank wells.
7. Incubation: Seal the plate with a sealing plate and incubate at 37°C for 30 minutes.
8. Washing: Carefully peel off the sealing plate film, discard the liquid, and dry it. Fill each well with washing liquid, leave it for 30 seconds, and discard it. Repeat this 5 times and pat dry.
9. Color development: Add the color developer A50 *μ*l to each well, and then add the color developer B50 *μ*l, mix gently by shaking, and develop the color at 37°C in the dark for 15 minutes.
10. Termination: Add 50 *μ*l of stop solution to each well to stop the reaction (blue to yellow currently).
11. Measurement: Zero the blank holes, and sequentially measure the absorbance (OD value) of each well at a wavelength of 450 nm.
12. Take the concentration of the standard as the abscissa and the OD value as the ordinate, draw a standard curve on the coordinate paper, and find out the corresponding concentration from the standard curve according to the OD value of the sample, and then multiply by the dilution factor.

### 2.5. Statistical Analysis

The SPSS13.0 statistical software was used to perform statistics on all data. Measurement data were expressed as mean ± standard deviation (). Comparisons between measurement data groups were performed by *t*-test, and comparisons between count data groups were performed by *χ*2 test. The relationship between age of onset, duration of disease, annual number of relapses, serum NMO-lgG concentration, and brain damage was analyzed by Logistic correlation coefficient. For each index, *α* = 0.05 was used as the test level, and *P* < 0.05 was considered statistically significant.

## 3. Results

### 3.1. Demographic and Clinical Manifestations of NMO and MS

According to the above criteria, 50 patients with NMO and 50 patients with MS were diagnosed. The general comparison between the two groups is shown in Table 1. Optic neuritis was the most common disease in NMO patients (73%), and another 5 patients occurred simultaneously. Myelitis (32%) is more common in MS patients than in NMO patients. Among them, 34 cases were positive for AQP4-IgG in NMO patients. In addition, the general clinical data showed that there was no significant difference in gender ratio, age of onset, course of disease, and annual recurrence between the two groups (*P* > 0.05). There were significant differences in the abnormal rate of head MRI and the number of immune diseases between NMO group and MS group (*P* < 0.05).

### 3.2. Head MRI Manifestations

The head MRI of patients with NMO and MS includes the sagittal and horizontal positions of T1WI, T2WI, and FLAIR sequences. Among them, the T2WI and FLAIR sequences are the clearest, showing long T2 signals, and long or slightly longer signals in T1WI sequence lesions. Of the 50 patients with NMO, MRI of the head was normal in 21 patients and abnormal imaging of the brain was found in 29 patients. 14 cases involved nonspecific lesions such as cerebral hemisphere. 5 cases involved the lateral ventricle, 8 cases around the third ventricle and the midbrain aqueduct, and 13 cases involved the brainstem (including 5 cases in the midbrain, 4 cases in the pontine, and 4 cases in the medulla oblongata). Two of the lesions were a direct extension of the medullary lesion to the high cervical spinal cord and involved the central canal, 4 of the thalamus (1 of which involved bilateral thalamus), 3 of the corpus callosum, 2 of the hypothalamus, and 2 of the cerebellum (the cerebellum). 1 case in the hemisphere and 1 case in the cerebellar arm were involved in both the supra- and under-the-scene). The above lesions were mostly abnormal signals in the form of spots, patches, and strips. The scan sequence of the head of the MS group was the same as that of the NMO. The MRI of the head of 50 patients was abnormal in 45 cases (abnormal rate 90%). Among them, 38 patients with MS had lateral ventricular lesions, and the others were hemiventricular lesions. The above lesions are mostly spot-shaped and patch-shaped abnormal signals, showing long T1 and long T2 abnormal signals, and FLAIR images show high signals.

### 3.3. MRI Findings of the Spinal Cord

The spinal cord lesions in 50 patients with NMO were all greater than or equal to 3 segments of which 30 (60%) involved the cervical spine, 8 (16%) involved the spinal cord, and 12 (34%) involved the cervical and thoracic spinal cord. It mainly involves the central gray and white matter of the spinal cord, and it shows plaques and line-like long T1 and long T2 signals. A total of 16 patients with MS had spinal MRI findings of which 14 were abnormal. There were 8 cases of cervical spinal cord involvement, 4 cases of thoracic spinal cord, and 2 cases of cervical spinal cord involvement. Its transection mainly involves white matter. The 14 spinal cord lesions were all less than 2 spinal cord segments, and the main manifestations were discontinuous patchy long T1 and long T2 signals. Swelling of the spinal cord can occur in the acute phase of patients with NMO and multiple sclerosis in the acute phase, but severe spinal atrophy can occur in the later stages of recurrent spinal cord disease in NMO patients.

### 3.4. Head MRI Enhanced Scan

**Figure 1:**
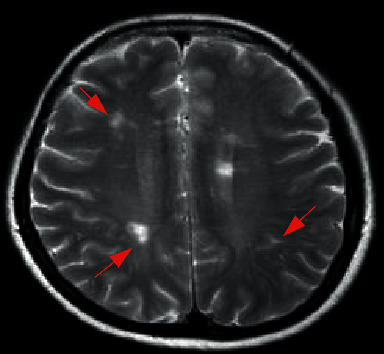
Female NMO patient, 60 years old. FLAIR cross section of the head shows multiple nonspecific focal lesions under the cerebral cortex.

**Figure 2:**
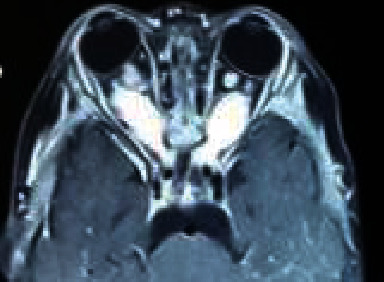
Female NMO patient, 25 years old, bilateral optic neuritis.

**Figure 3:**
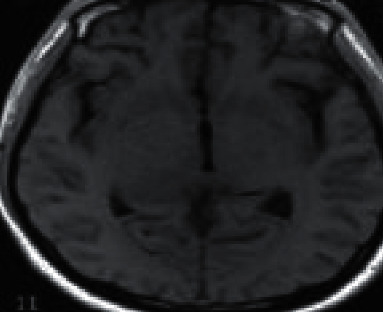
Female NMO patient, 57 years old, with midbrain and optic cross lesions.

**Figure 4:**
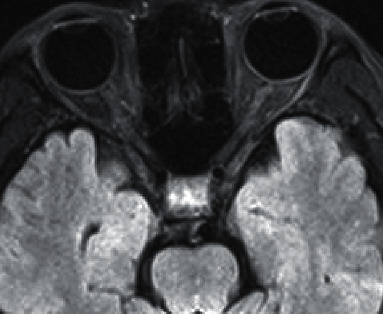
Female NMO patient, T2WI scan of 37-year-old cervical spinal cord cross section shows cervical spinal swelling, and the lesion mainly involves central gray matter of the spinal cord.

**Figure 5:**
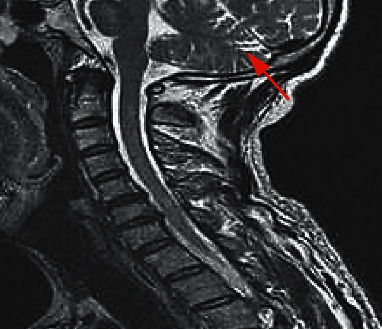
Female NMO patient, 44 years old, with a cross section of the head FLAIR showing a large lesion in the posterior horn of the right ventricle and involving the thalamus.

**Figure 6:**
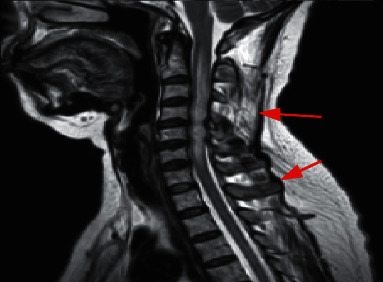
Female NMO patient, 45 years old, with sagittal T2WI showing a medullary and cervical spinal cord lesion with swelling in the spinal cord.

Among the 29 patients with NMO head lesions, 8 patients with intracranial lesions underwent enhanced MRI scans of the head. The results of the enhanced scans showed that 12.5% (1/8) of patients had significant enhancement of intracranial lesions, which were mainly manifested as sheet enhancement. In contrast, 61.54% (8/13) patients with MS had abnormal MRI enhancement scans on their heads, which were mainly manifested as irregular patches, rounds, and round-like enhancements (Table 2). Among them, Figures 1–6 were typical MRI images of 29 patients with NMO head lesions.

### 3.5. Analysis of Related Risk Factors Such as Intracranial Lesions and AQP4 Antibody Concentration in NMO Patients

**Figure 7:**
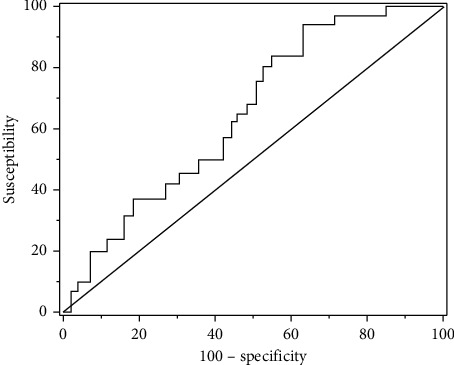
ROC curve.

The 34 AQP_4_-IgG positive patients had an AQP_4_-IgG concentration of 390.47 ± 169.21 ng/L. Logistic regression analysis was performed with age at onset, duration of disease, annual relapse rate, and AQP_4_-IgG concentration as independent variables, and the presence or absence of brain lesions as dependent variables. The results showed that gender, duration of disease, annual relapse rate, and AQP_4_-IgG concentration were not related to brain lesions in NMO patients, and the age of onset was included in the regression equation (Table 3). The OR value of 1.104 indicated that the age of onset and brain lesions are certainly related. The predictive value of AQP4-IgG for disease recurrence in NMO patients was further analyzed.  Figure 7 showed the ROC curve. It suggested that there was a positive correlation between the expression of serum AQP4 and recurrence in NMO patients. It showed that the expression of serum AQP4-IgG in patients with NMOSDs was closely related to the severity of the disease, prognosis, and recurrence, and could predict the recurrence of NMO patients.

## 4. Discussion

Previous studies have suggested that NMO mainly affects the optic nerve and spinal cord, but the brain is not involved [11]. In recent years, with the widespread application of MRI, more and more studies have found that lesions can occur in the brain of NMO patients. Because the clinical manifestations of NMO and MS are similar, the optic nerve, spinal cord, and brain can be involved in imaging. Therefore, it is of great significance to understand the MRI manifestations of NMO brain [12, 13]. The clinical, brain imaging, and NMO-IgG characteristics of NMO and MS patients in the two groups were analyzed. The results showed that both groups had gender ratio, abnormal brain MR1 expression, serum AQP_4_-IgG-positive, and other immune diseases and symptoms. Statistical differences (*P* < 0.05) indicate that NMO and MS have different clinical and imaging manifestations, and serum NMO-IgG levels are different.

Among the 29 abnormal head lesions in NMO patients, nonspecific lesions were the most common. The lesions were mostly small degeneration lesions in the cortex, near the subcortex and deep white matter, which showed spotted, patchy abnormal signals, single or multiple, slightly higher or higher signal in T2WI or FLAIR phase. According to relevant literature reports, this type of lesions may be due to the late onset of NMO and the older age of the onset of age-related ischemic lesions [14]. However, for younger NMO patients and patients without high-risk factors for brain lesions, this disease characteristic cannot be fully explained, and the cerebral cortex is also the region of high AQP_4_ expression. According to reports by Pittock et al., patients with normal MRI scans of the skull at the onset of NMO have a higher probability of various types of lesions including nonspecific, atypical lesions as the disease progresses. Therefore, its formation may also be related to the patient's disease progression and cerebral cortex AQP_4_ expression, but the specific formation mechanism needs to be further studied. Among the 29 patients with NMO, 8 cases were paraventricular and midbrain aqueducts, 4 were thalamus, 2 were hypothalamus, and 13 were brain stem. In addition, 2 cases involving the brainstem involved the central tube of the bulbus. Therefore, the number of cases around the third ventricle and midbrain aqueduct and the central tube of the bulbus was 10. Among them, the number of cases involving the tertiary and midbrain aqueducts, around the central duct, hypothalamus, and brainstem was statistically significant compared with the MS group (*P* < 0.05).

In the present study, there were no lower line-like high signals in 3 cases of corpus callosum lesions in the NMO group, which were mainly manifested by T2WI multiple sheet abnormal signals, which were difficult to distinguish from MS in the shape of the lesions. In addition, the lesions are not statistically significant, which is related to the lower number of positive cases in the corpus callosum of the NMO. This may be related to the MRI scan method of the head. Currently, the internationally recommended thin-layer sagittal FLAiR image of the corpus callosum can significantly improve the corpus callosum positive rate [15, 16]. At the same time, studies have shown that there is a significant difference between corpus callosum diffusion tensor analysis and MS in NMO patients, which can be used to distinguish between the two. A total of 8 patients underwent head enhancement scans in NMO patients, but only 1 patient had significantly enhanced head lesions, and 61.54% (8/13) of MRI enhancement scans of MS patients had abnormal enhancement. At present, domestic researches tend to focus on the intensive brain lesions in NMO patients. However, Krampla et al. found that NMO lesions can be enhanced, but whether brain lesions are enhanced may be related to drug treatment, the time of onset, and the interval between MRI measurements [17]. This study shows that brain lesions in some patients with NMO can be enhanced, but there are differences in the enhancement rates between NMO and MS patients, which may be related to the different pathogeneses.

In the correlation analysis using brain lesions as the dependent variable, we can see that the age of onset of NMO, course of disease, gender, annual relapse rate, and AQP_4_-IgG concentration have no correlation with brain lesions in NMO patients, but the age of onset and brain. The OR value of the lesion was 1.104. Although there was no statistical significance (*P* > 0.05), it showed that the correlation between the two was greater, and that as the age of onset increased, the more likely the brain lesions appeared. According to research, the age of onset is related to the course of NMO recurrence. The later the onset, the more frequent the recurrence, and therefore, the greater the likelihood of brain damage. In addition, it is worth noting that domestic and foreign studies have shown that 10% to 20% of NMO patients are recurrent, and after recurrence, lesions meeting the Barkhof criteria may appear in the skull. These lesions are mainly peripheral ventricle lesions [18]. After repeated attacks, the brain lesions of NMO and MS have overlapped and become difficult to distinguish, which leads to diagnostic errors. Therefore, in clinical work, we should try to distinguish the two diseases as much as possible, so that patients get treatments different from MS.

## 5. Conclusion

NMO is a disease of the central nervous system that is mainly affected by the optic nerve and the spinal cord simultaneously or successively, but brain damage is also very common, accounting for more than half. Therefore, the following conclusions can be drawn from this exploration: (1) NMO diagnosis should not be excluded for patients with internal lesions, especially those with head lesions that do not meet the performance of MS. (2) Patients with increased paraventricular lesions accompanied by optic neuritis and myelitis should conduct AQP4-IgG and MS detection as much as possible. (3) NMO diagnosis should be considered for lesions with high expression of AQP4, such as the third ventricle, midbrain aqueduct, and brainstem, as well as patients with optic neuritis and myelitis. (4) The later the onset age is, the greater the possibility of brain injury is.

Of course, there are still some research deficiencies. First, the selected research objects are from our hospital, and the research results may be biased. More detailed research with multicenters and large sample size needs to be conducted in the future. Next, the correlation between serum AQP4 and intracranial lesions in NMO patients is mainly analyzed. In the follow-up study, more indexes such as IL-6 and IL-27 can be further detected to have a clearer understanding of their correlation with the long-term prognosis and recurrence of patients, and finally improve the clinical detection methods.

## Figures and Tables

**Table 1:** Comparison of clinical data of NMO and MS patients.

	NMO	MS
People	50	50
*General clinical data*		
Gender ratio (M/F)	01 : 03.5	01 : 01.4
Age of onset (years)	37 ± 16.79	35 ± 9.97
Course of disease (year)	3.3 ± 5.63	1.5 ± 3.41
Annual relapses	1.27 ± 0.96	1.41 ± 0.81
*Onset form*:		
Acute myelitis (case)	12	16
Acute optic neuritis (example)	33	34
AQP_4_-IgG (+)	34	1
Head MRI abnormal rate	29/50 ^*∗*^	45/50
Onset with immune disease	13 ^*∗*^	0

*Note*. *∗* indicates that compared with MS group, *P* < 0.05.

**Table 2:** Comparison of head and neck MRI in NMO and MS patients (*n*/%).

Lesion site	NMO	MS	*χ*2	*P*
Around the lateral ventricle	5 (17.24)	38 (84.44)	32.718	<0.05
Peripheral and midbrain aqueducts and central bulbs of the medulla oblongata	10 (34.48)	0 (0.00)	—	<0.05
Thalamus	4 (13.79)	2 (4.44)	—	>0.05
Brain stem	13 (44.83)	10 (22.22)	4.207	<0.05
Carcass	3 (10.34)	3 (6.67)	—	>0.05
Hypothalamus	2 (6.90)	0 (0.00)	—	<0.05
Cerebellar involvement	2 (6.90)	3 (6.67)	—	>0.05
Brain stem extends to cervical spinal cord	2 (6.90)	0 (0.00)	—	<0.05
Cervical spinal cord abnormality	30/29	8/14	—	>0.05

**Table 3:** Parameter estimation and test results of the logistic regression model.

Variable	Degrees of freedom	Partial regression coefficient	*χ*2	*P*	OR
Constant term	1	−4.228	3.897	0.46	-
Age of onset	1	0.095	3.074	0.07	1.104

## Data Availability

The data used to support the findings of this study are available from the corresponding author upon request.
